# Rehabilitation versus autologer Ersatz in der postakuten Phase nach Ruptur des vorderen Kreuzbandes: Evidenzbasierte Kommentierung der multizentrischen randomisierten ACL-SNNAP-Studie

**DOI:** 10.1007/s00132-023-04366-6

**Published:** 2023-05-23

**Authors:** Thoralf Randolph Liebs, Luzi Dubs, Dirk Stengel, Tobias Renkawitz, Arbeitsgruppe Evidenzbasierte Medizin der Deutschen Gesellschaft für Orthopädie und Unfallchirurgie

**Affiliations:** 1 Klinik für Orthopädie und Traumatologie, Zentrum für muskuloskelettale Infektionen, Universitätsspital Basel, Spitalstrasse 21, Basel, Schweiz; 2 Klinik für Orthopädie und Traumatologie, Kantonsspital Aarau, Aarau, Schweiz; 3 Winterthur, Schweiz; 4 BG Kliniken – Klinikverbund der gesetzlichen Unfallversicherung gGmbH, Berlin, Deutschland; 5 Orthopädische Universitätsklinik Heidelberg, Ruprecht-Karls-Universität, Heidelberg, Deutschland

## Originalpublikation

Beard DJ, Davies L, Cook JA et al (2022) Rehabilitation versus surgical reconstruction for non-acute anterior cruciate ligament injury (ACL SNNAP): a pragmatic randomised controlled trial. Lancet 400(10352):605–615.

## Hintergrund.

Rupturen des vorderen Kreuzbandes (VKB) zählen international zu den häufigsten Verletzungen des Bewegungsapparates.

Das primär mechanistische Ziel der Therapie ist die Stabilisierung des Kniegelenks, um Betroffenen die Wiedererlangung ihres ursprünglichen physischen Aktivitätsniveaus zu ermöglichen. In einer eher biologischen Denkschule wird davon ausgegangen, dass die natürlichen Heilungsvorgänge ab dem Zeitpunkt des Unfalls, unterstützt durch Physiotherapie, ausreichend sind, um zu vergleichbaren Behandlungsresultaten zu kommen.

Bislang ist nicht eindeutig geklärt, ob die rasche Restitution der körperlichen Leistungsfähigkeit besser durch eine nichtoperative oder operative Behandlung erreicht werden kann – die Studienlage ist widersprüchlich oder unzureichend. Gerade in der postakuten, chronischen Phase einer Kniegelenkinstabilität aufgrund einer VKB-Ruptur besteht unverändert therapeutische Unsicherheit (Equipoise), wann ein konservatives Vorgehen, bestehend aus Physiotherapie, eingeleitet bzw. fortgeführt werden sollte, oder wann eine arthroskopisch assistierte VKB-Ersatzoperation empfohlen werden sollte.

Beard und Mitarbeiter haben sich in der hier kommentierten Studie der Frage gestellt, ob bei Patient(inn)en mit symptomatischer VKB-Insuffizienz nach Abklingen der akuten Phase (in der Regel nach etwa 6–9 Monaten) ein nichtoperatives oder ein operatives Vorgehen klinisch bessere Ergebnisse erzielen kann [1].

## Methode.

Zur Beantwortung dieser Fragestellung führten die Autoren eine multizentrische randomisierte Studie (ACL Surgery Necessity in Non-Acute Patients, SNNAP Trial) an 29 Zentren im Vereinigten Königreich durch (Tab. 1). Nach einer Pilotuntersuchung wurde das RCT prospektiv in clinicaltrials.gov registriert (NCT02980367) und das Studienprotokoll [2] zusammen mit dem statistischen Auswertungskonzept [5] vorab veröffentlicht.

Eingeschlossen wurden Patient(inn)en mit symptomatischer Insuffizienz (entweder Teil- oder Totalruptur) des nativen vorderen Kreuzbandes (Instabilitätsepisoden mit „giving-way“ oder Instabilitätsgefühl), bei denen die Diagnose durch klinische Untersuchung und MRT bestätigt wurde. Es wurde nicht zwischen subjektiv empfundener und objektiv gemessener Instabilität unterschieden. Der pragmatische Ansatz erlaubte alle lokal etablierten Therapie- und Rehabilitationsstandards. Operateure mussten ihre Erfahrung durch wenigstens 50 VKB-Ersatzplastiken nachweisen. Es wurden sowohl „bone-tendon-bone“ (BTB) als auch Hamstring-Transplantate gestattet.

Die Fallzahlplanung für das Hauptzielkriterium, der Gruppendifferenz im Knee Injury and Osteoarthritis Outcome Score (KOOS4) 18 Monate nach Randomisierung, beruhte auf der realistischen und durch Vorinformationen gesicherten Annahme einer minimal klinisch relevanten Differenz (MCID) von 8,0 Punkten bei einer Standardabweichung von 19,0; einer Power von 90 % und einem akzeptierten zweiseitigen Fehler erster Art von 5 %. Demgemäß sollten auswertbare Daten von 320 Patient(inn)en vorliegen.

Neben der Intent-to-Treat(ITT)-Analyse, bei der Teilnehmende gemäß ihrer Zufallszuordnung ausgewertet wurden, erfolgten auch Per-Protokoll (PP) Analysen. Die konservative PP-Analyse schloss alle Patient(inn)en aus, welche die Anforderungen der Studie für jede im Protokoll angegebene Intervention nicht erfüllten. Die pragmatische unterschied sich von der konservativen PP-Analyse dadurch, dass Proband(inn)en nicht ausgeschlossen wurden, wenn sie eine unzureichende Physiotherapie erhalten oder diese nicht abgeschlossen hatten (was nach klinischer Erfahrung vorkommen kann). Zudem wurde die Fläche unter der Kurve („area under the curve“ [AUC]) als zeitabhängige Größe ermittelt.

Als sekundäre Endpunkte wurden die Aktivitätsskala nach Tegner, sowie Fragebögen zur gesundheitsbezogenen Lebensqualität (EuroQoL-5D, ACL QOL) und das Auftreten von Komplikationen erhoben.

Zwischen dem 01.02.2017 und 12.04.2020 wurden 1403 Patient(inn)en gescreent, von denen 485 die Teilnahme verweigerten. Die geeigneten Proband(inn)en wurden per Web-basierter Randomisierung im 1:1-Verhältnis, mit variabler Blockgröße und Stratifizierung nach Studienzentrum und dem KOOS4-Ausgangswert, entweder der primär konservativen (*n* = 160) oder der operativen Gruppe (*n* = 156) zugewiesen. Mit 316 Teilnehmenden wurde die angestrebte Fallzahl knapp unterschritten. Eine Verblindung auf Patientenebene wäre nur mit sehr hohem Aufwand möglich gewesen und wurde daher nicht durchgeführt.

Ein CONSORT-Flussdiagramm liegt vor, und Details der Run-in-Phase wurden im elektronischen Appendix ausführlich beschrieben.

Schon im Studienprotokoll wird dabei aufgeführt, dass in der konservativen Gruppe eine erste Bewertung nach 3 Monaten erfolgen sollte, um den Erfolg der konservativen Therapie einzuschätzen. Sofern kein Therapieerfolg zu verzeichnen war, bestand die Möglichkeit der kurzfristigen operativen Intervention. Diese Therapieänderung, die bei 39 (24 %) Teilnehmenden der Rehabilitationsgruppe erfolgte, wurde nicht als Cross-Over gewertet. Hingegen wurden 43 von 156 Patient(inn)en (28 %) der ursprünglich „operativen Gruppe“ de facto nicht operiert. Diese Möglichkeit wurde im Studienprotokoll jedoch nicht antizipiert, sodass diese Patient(inn)en als Cross-Over gelten sollten.

Der KOOS4 18 Monate nach Randomisierung wies in der Rekonstruktionsgruppe bessere Werte auf (73,0 [SD 18,3]) als in der Rehabilitationsgruppe (64,6 [SD 21,6]). Die unadjustierte Mittelwertdifferenz betrug 8,3 (95 % Konfidenzintervall [KI] 3,3–13,3), die adjustierte Mittelwertdifferenz 7,9 (95 % KI 2,5–13,2) Punkte.

**Figure 1:**
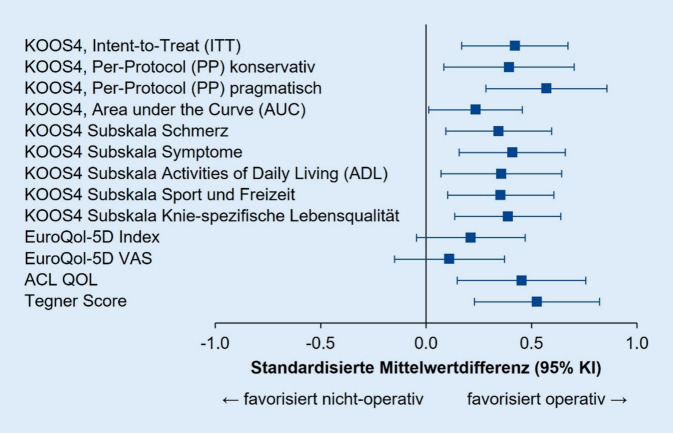

Abb. 1 gibt einen Überblick über die mittels STATA 16.1 nachberechneten standardisierten Mittelwertdifferenzen (SMD) mit 95 % KI entsprechend der publizierten Daten. Diese entsprechen näherungsweise den Effektstärken. Tegner-Scores wurden als Median mit Interquartilspanne (IQR) berichtet. In Anlehnung an die im Cochrane Handbook empfohlenen Approximationen (https://handbook-5-1.cochrane.org/chapter_7/7_7_3_5_mediansand_interquartile_ranges.htm) wurden Mediane mit Mittelwerten gleichgesetzt und die Standardabweichung als IQR/1,35 geschätzt.

In der operativen Gruppe erlangten 27/95 (28 %), in der nichtoperativen Gruppe 21/86 (24 %) ihr ursprüngliches körperliches Leistungsniveau. Dies entspricht einem relativen Risiko von 0,86 (95 % KI 0,53–1,40) und einer absoluten Risikodifferenz von 4,0 (−8,8 bis 16,8 %) und war damit mit dem Zufall vereinbar. Komplikationen traten bei 10/156 (6,4 %) Patient(inn)en nach Kreuzbandersatz und 11/160 (6,9 %) Patient(inn)en im Kontrollarm auf.

Die Autoren schlussfolgern, dass bei Patient(inn)en mit einer nichtakuten VKB-Verletzung, welche eine persistierende symptomatische Instabilität aufweisen, ein chirurgisches Management der Rehabilitation überlegen ist.

## Kommentar

Die Autoren adressieren eine klinisch wichtige und häufig debattierte Fragestellung. Das gewählte randomisierte Design ist geeignet, um die Studienhypothese zu beantworten. Der multizentrische Ansatz wird genutzt um eine hohe externe Validität zu erzielen, sodass die Ergebnisse auf die Gesamtheit der Patient(inn)en, welche die Einschlusskriterien erfüllen, übertragbar sind.

Hochrangige „general medical journals“ tendierten in der Vergangenheit dazu, negative Ergebnisse einer operativen Intervention in Orthopädie und Unfallchirurgie hervorzuheben [4]. Daher ist eine Publikation im *Lancet,* bei der eine Überlegenheit einer operativen Intervention gegenüber einer nichtoperativen Behandlungsstrategie beschrieben wird, von besonderem Interesse.

Die ACL-SNNAP-Studie weist eine Vielzahl von Stärken auf. Neben dem angemessenen Studiendesign und dem multizentrischen Ansatz wurde die Studie vorab registriert und das Studienprotokoll und das statistische Auswertungskonzept veröffentlicht – mehr Transparenz in der Planung einer klinischen Studie ist kaum denkbar. Die Fallzahlenberechnung beruhte auf realistischen Annahmen, und die angestrebte Stichprobengröße wurde nahezu erreicht. Die primäre Zielvariable (KOOS4) ist patientenzentriert. Um das Problem des Score-Bias zu adressieren, sind Beard et al. der Empfehlung der KOOS-Autoren gefolgt und haben auch eine Analyse der Subskalen des KOOS vorgelegt. Diese hat keine Abweichung der beobachteten Effekte gezeigt. Als besonders clever ist hervorzuheben, dass die Autoren schon bei der Studienplanung das Problem berücksichtigt haben, dass innerhalb des nichtoperativen Studienarms ein Cross-Over zur operativen Therapie zu erwarten ist.

Die nachberechneten standardisierten Mittelwertdifferenzen liegen im Median bei 0,39 (Spanne 0,11–0,57) und weisen damit auf einen moderaten Effekt der chirurgischen Intervention hin. Alle primären und sekundären Endpunktanalysen favorisieren die Kreuzbandersatzoperation. Unter Zugrundelegung der Eckdaten des britischen Gesundheitssystems wurde bei einer „willingness-to-pay“ pro „quality-adjusted life year“ (QALY) von £ 30.000,00 eine Wahrscheinlichkeit von 72 % ermittelt, dass die chirurgische Option kosteneffektiv ist. Derartige Ansätze spielen im deutschen Gesundheitssystem (noch) keine Rolle, und ein arthroskopisch assistierter VKB-Ersatz schlägt hierzulande, je nach Art des gewählten Transplantates, stationärer Behandlung usw. mit tangiblen Kosten von € 5000,00–10.000,00 zu Buche.

Allerdings weist auch die zunächst als durchdacht erscheinende ACL-SNNAP-Studie Schwächen auf. So war im Studienprotokoll z. B. nicht vorgesehen, dass Patient(inn)en (hier: 43/156) zur operativen Therapie randomisiert wurden, aber keine Operation unterliefen. Im britischen Gesundheitssystem sind Wartezeiten nicht ungewöhnlich – 11 Teilnehmende warteten vergeblich auf einen Operationstermin. Auf der anderen Seite wurden 39 Patient(inn)en von den 160 Teilnehmenden des nichtoperativen Arm dann doch operiert. Hier ist denkbar, dass sich physisch aktivere und ungeduldigere Teilnehmende eher für eine baldige Operation entschieden. Somit war hier ein gewisser Selektions-Bias in der Run-in-Phase vorprogrammiert, welcher auch nicht durch statistische Analysen ausgeglichen werden konnte.

Angesichts dieses doch erheblichen Cross-Over zwischen beiden Gruppen wäre die Gruppenzugehörigkeit am ehesten mit „Initiale Empfehlung VKB-Plastik“ vs. „Initiale Empfehlung Rehabilitation“ zu charakterisieren.

Patient(inn)en in der Rehabilitationsgruppe erhielten sechs Therapiesitzungen über einen Zeitraum von 3 Monaten. Hier stellt sich die Frage, ob zwei Behandlungen pro Monat über einen Zeitraum von 3 Monaten für dieses Krankheitsbild ausreichend sind. Die Anzahl der Therapiesitzungen in der chirurgischen Gruppe wurde nicht beschrieben, obgleich der postoperative Therapieplan über 9 Monate genau definiert wurde. Es könnte sich der Eindruck aufdrängen, dass operierte Patient(inn)en länger Physiotherapie als Patient(inn)en der Rehagruppe erhielten.

Die Follow-up-Frequenz nach 18 Monaten liegt bei 79 % und damit knapp unter dem Schwellenwert von 80 %, welcher üblicherweise für eine robuste Nachuntersuchung angelegt wird.

In einem optimalen Studiendesign hätte eine Verblindung dergestalt stattgefunden, dass alle potenziell geeigneten Patient(inn)en verblindet eine Verum- oder Scheinoperation (z. B. Hautinzision einschl. der Transplantatentnahmestellen) unterlaufen hätten und auch bei einer körperlichen Nachuntersuchung unabhängige verblindete Untersucher durch Pflastermaskierung des Kniegelenks keine Rückschlüsse auf die erfolgte Therapie hätten ziehen können. Eine derartige Perfektion erfordert jedoch immense Ressourcen und wird erfahrungsgemäß auch nicht ohne Rückfragen von einer Ethikkommission positiv beraten werden.

Allerdings hätte die statistische Analyse verhältnismäßig einfach verblindet durchgeführt werden können, und idealerweise wäre das Autorengremium erst nach der Bewertung der Ergebnisse der statistischen Analyse entblindet worden.

Die Forschergruppe wählte ein pragmatisches Design, bei dem möglichst wenige an den teilnehmenden Zentren etablierten Standards harmonisiert werden müssen. Allerdings sind pragmatische Designs v. a. dann angezeigt, wenn Tausende Betroffene eingeschlossen werden sollen. Im konkreten Fall war es aufgrund der angestrebten Stichprobe weder erforderlich noch sinnvoll, die Anzahl von Expositions- und Outcome-Variablen und damit ihre präzise Operationalisierung zu limitieren. Die „Table 1“ der Originalpublikation, welche klassischerweise das Basisprofil einer Studie illustriert, beinhaltet als demografische Indikatoren lediglich Alter und Geschlecht.

Es wird nicht dargestellt, wie viele Patient(inn)en der operativen Gruppe ein BTB- und wie viele ein Hamstring-Transplantat erhielten. Da in der Literatur unterschiedliche Therapieeffekte zwischen diesen Techniken beschrieben worden sind, ist dieser Punkt relevant [3]. Da dies naturgemäß nur die chirurgische Gruppe betrifft, konnte auch keine Gleichverteilung durch Randomisierung ermöglicht werden.

Wie bei einer Studie dieser Größenordnung zu erwarten, scheint es auch in dieser Studie Rekrutierungsprobleme gegeben zu haben: trotz Beteiligung von 29 Zentren bedurfte es einer Rekrutierungsdauer von 38 Monaten, um 316 Patient(inn)en zu akquirieren. Dies bedeutet, dass ca. ein Patient pro Studienzentrum pro Monat gescreent wurde und nur alle 7 Monate ein Patient pro Studienzentrum operiert wurde. Dem Appendix ist zu entnehmen, dass ein großes Ungleichgewicht zwischen den Studienzentren hinsichtlich der Patientenrekrutierung bestand. So haben beispielsweise fünf Studienzentren nur einen oder zwei Patient(inn)en rekrutiert, während zwei andere Studienzentren zusammen ein Viertel aller Patient(inn)en rekrutiert haben.

Es fällt schwer einzuschätzen, ob aus all diesen Problemen ein systematischer Fehler (Bias) resultiert, welcher die Studienergebnisse verfälscht haben könnte. Jedenfalls fällt auf, dass die Baseline-Werte sich hinsichtlich der Hauptzielvariable KOOS4 zwischen den Gruppen leicht unterscheiden, wobei die Patient(inn)en der operativen Gruppe um 2,4 Punkte bessere Werte aufweisen. Dieses könnte als Hinweis auf Probleme bei der Randomisierung gewertet werden. Allerdings weisen die Werte bei dem – allerdings krankheitsunspezifischen – EQ-5D-5L VAS in die entgegengesetzte Richtung, was gegen diesen Verdacht sprechen würde. Wenn nun allerdings zur Berechnung des Therapiegewinns der bei der Randomisierung beobachtete Gruppenunterschied von 2,4 Punkten für die Hauptzielvariable von dem beobachteten Gruppenunterschied zum Zeitpunkt des 18-Monats-Follow-up subtrahiert werden würde, dann würde der Gruppenunterschied unter die Grenze des klinisch wichtigen Unterschiedes fallen.

## Fazit

Zusammenfassend handelt es sich beim ACL-SNNAP-Trial um eine nach heutigem Kenntnisstand sehr gut geplante, durchgeführte und transparent berichtete Studie. Insbesondere die vorab definierte Handlungsanweisung, wie mit dem zu erwartenden Cross-Over von der Rehabilitationsgruppe in die chirurgische Gruppe umgegangen werden soll, war vorausschauend und clever.

Bei vertiefter Betrachtung kommen jedoch einige Mängel ans Licht. So wird beispielsweise der Randomisierungseffekt durch Nachselektion verwässert, sodass bei den Interventionen am ehesten von initialer Empfehlung VKB-Plastik vs. initialer Empfehlung Rehabilitation gesprochen werden kann. Des Weiteren gab es Rekrutierungsprobleme, die übliche Follow-up-Quote von 80 % wurde nicht erreicht und die statistische Analyse ist nicht verblindet erfolgt.

Insgesamt treten unseres Erachtens jedoch die methodischen und analytischen Schwächen hinter den Stärken zurück, auch wenn die Ergebnisse nicht unkritisch in den Versorgungsalltag überführt werden dürfen. Es sei angemerkt, dass es sich hier um eine der wenigen Veröffentlichungen in einem hochrangigen allgemeinmedizinischen Journal handelt, in welcher auf orthopädischem/unfallchirurgischem Fachgebiet eine operative Intervention favorisiert wird.

Zusammenfassend liefert diese Studie Hinweise darauf, dass Patient(inn)en mit einer nichtakuten VKB-Verletzung und persistierender symptomatischer Instabilität ein chirurgischer VKB-Ersatz gegenüber einer alleinigen Physiotherapie empfohlen werden sollte. Folgestudien sollten die hier identifizierten Mängel beheben, damit diese wertvolle Fragestellung mit noch größerer Sicherheit beantwortet werden kann.

**Table 1:** 

S	Studiendesign	Randomisiert, multizentrisch, nicht verblindet
P	Patient(inn)en (Ein- und Ausschlusskriterien)	316 Patient(inn)en aus 29 Zentren im Vereinigten Königreich *Einschlusskriterien* Symptomatische Insuffizienz (entweder Teil- oder Totalruptur) des nativen vorderen Kreuzbandes (Instabilitätsepisoden mit „giving-way“ oder Instabilitätsgefühl) bestätigt durch klinische Untersuchung und MRT Patient(in) willigt zur Teilnahme an der Studie ein Alter ab 18 Jahre *Ausschlusskriterien* Frühere Kreuzbandrekonstruktion des betroffenen Kniegelenks Akute Phase der primären Verletzung des VKB (Patient(in) hat sich noch nicht von den akuten Symptomen der initialen VKB-Verletzung erholt) Voroperationen am betroffenen Kniegelenk (gestattete Ausnahmen: diagnostische Arthroskopie oder Meniskusteilentfernung) oder gleichzeitige schwere Verletzung des kontralateralen Kniegelenks Meniskusbeschwerden, die eine sofortige Operation rechtfertigen würden (blockiertes Kniegelenk, großer Korbhenkelriss mit mechanischen Symptomen) Gonarthrose, Kellgren und Lawrence Skala Grad 3 oder 4 Verletzung eines der Kollateralbänder Grad 3, des HKB oder eine Verletzung der posterolateralen corner Entzündliche Gelenkerkrankung Schwangerschaft
I	Intervention	Operatives Management (VKB-Plastik, entweder mittels BTB oder Hamstring), *N* = 156 oder Nichtoperatives Vorgehen (Rehabilitation/Physiotherapie, Minimum 6 Sitzungen binnen 3 Monaten), *N* = 160
O	Outcome	*Primär* Knee Injury and Osteoarthritis Outcome Score (KOOS4) 18 Monate nach Randomisierung (operativ 73,0 ± 18,3; nichtoperativ 64,6 ± 21,6) *Sekundär* Modifizierter Tegner-Score, EuroQol-5D‑5, Subskalen des KOOS4, Anterior Cruciate Ligament Quality of Life Score (ACL-QOL), Kosteneffektivität („costs per QALY“)
N	Nutzen	Bei Patient(inn)en mit einer nichtakuten VKB-Verletzung mit persistierender symptomatischer Instabilität ist ein chirurgisches Management im Vergleich zur Rehabilitation beim 18-Monate-Follow-up klinisch überlegen und kosteneffektiver
